# Robert B. Sim—Tribute

**DOI:** 10.3390/v13091681

**Published:** 2021-08-25

**Authors:** Anna Erdei

**Affiliations:** 1 Department of Immunology, Eötvös Loránd University, 1053 Budapest, Hungary; anna.erdei@ttk.elte.hu; 2 MTA-ELTE Immunology Research Group, Eötvös Loránd University, 1053 Budapest, Hungary

I met Bob in 1985, when I moved with my family to Oxford for a two-year EMBO fellowship at the MRC Immunochemistry Unit. My previous methodological experience was mainly related to cells—I carried out functional studies using macrophages and lymphocytes. Working with Bob provided me with the opportunity to acquire biochemical methods at the highest level, including isolation of complement proteins, radioiodinating cells, running immunoprecipitates on SDS-PAGE and identifying complement receptors on immune cells.

Bob was not only a prolific molecular immunologist, mentor to students and fount of knowledge for colleagues, he was also always generous and encouraging when giving help and advice in the lab. The door of his small office crowded with reprints and notes was always open, and he was very much involved in the benchwork of everyone in the lab. He was a marvellous companion and friend who readily shared his precious chemicals and reagents with everyone even after leaving his lab.

I recall the warm hospitality that he provided with his wife Edith and children Grace and Francis in their home. Bob and Edith, together with Ken Reid and Duncan Campbell provided a special “Scottish atmosphere” in the unit, which I enjoyed a lot. Each year the whole lab was ritually invited to a Burns supper to eat haggis and drink whisky. I vividly remember these very special moments which we enjoyed with our families. Bob was born in Crieff where the spirit of the nearby world-famous Innerpeffray Library may have given an impetus to his journey in the academic world. At the beginning of his scientific journey, he was privileged to work with Nobel laureate Professor Rodney Porter.

Bob’s research ensued a number of breakthroughs in the field of complement which are highly quoted and provided valuable sources of new directions. I am indebted for the opportunity to work with him.

He kindly reviewed and commented on one of our recent manuscripts, which we dedicate to his memory.

